# Discovery of Urinary Proteomic Signature for Differential Diagnosis of Acute Appendicitis

**DOI:** 10.1155/2020/3896263

**Published:** 2020-04-04

**Authors:** Yinghua Zhao, Lianying Yang, Changqing Sun, Yang Li, Yangzhige He, Li Zhang, Tieliu Shi, Guangshun Wang, Xuebo Men, Wei Sun, Fuchu He, Jun Qin

**Affiliations:** ^1^School of Life Sciences, Peking University, Beijing 100871, China; ^2^State Key Laboratory of Proteomics, Beijing Proteome Research Center, National Center for Protein Sciences (Beijing), Beijing Institute of Lifeomics, Beijing 102206, China; ^3^Joint Center for Translational Medicine, Tianjin Baodi Hospital, Tianjin 301800, China; ^4^Central Research Laboratory, Peking Union Medical College Hospital, Chinese Academy of Medical Sciences & Peking Union Medical College, Beijing 100110, China; ^5^Center for Bioinformatics, East China Normal University, Shanghai 200241, China; ^6^Alkek Center for Molecular Discovery, Verna and Marrs McLean Department of Biochemistry and Molecular Biology, Department of Molecular and Cellular Biology, Baylor College of Medicine, Houston, Texas 77030, USA

## Abstract

Acute appendicitis is one of the most common acute abdomens, but the confident preoperative diagnosis is still a challenge. In order to profile noninvasive urinary biomarkers that could discriminate acute appendicitis from other acute abdomens, we carried out mass spectrometric experiments on urine samples from patients with different acute abdomens and evaluated diagnostic potential of urinary proteins with various machine-learning models. Firstly, outlier protein pools of acute appendicitis and controls were constructed using the discovery dataset (32 acute appendicitis and 41 control acute abdomens) against a reference set of 495 normal urine samples. Ten outlier proteins were then selected by feature selection algorithm and were applied in construction of machine-learning models using naïve Bayes, support vector machine, and random forest algorithms. The models were assessed in the discovery dataset by leave-one-out cross validation and were verified in the validation dataset (16 acute appendicitis and 45 control acute abdomens). Among the three models, random forest model achieved the best performance: the accuracy was 84.9% in the leave-one-out cross validation of discovery dataset and 83.6% (sensitivity: 81.2%, specificity: 84.4%) in the validation dataset. In conclusion, we developed a 10-protein diagnostic panel by the random forest model that was able to distinguish acute appendicitis from confusable acute abdomens with high specificity, which indicated the clinical application potential of noninvasive urinary markers in disease diagnosis.

## 1. Introduction

Acute appendicitis is one of the most common surgical emergencies in clinic worldwide [1]. However, the precise diagnosis of acute appendicitis is still a challenge, especially in children and childbearing women. The absence of classical clinical signs presents in 30~45% of patients [2]. Also, similar symptoms of other acute abdominal inflammations (such as gastrointestinal disorders, cholelithiasis, and gynecological diseases) with appendicitis lead to misdiagnosis and high negative appendectomy rates, which is reported to a range between 10% and 32% [3–5]. In addition, the missed diagnostic rate of acute appendicitis ranges from 11% to 28% [6, 7], and the delays in diagnosis and treatment would lead to the increased risk of appendix rupture, abscess, and even septicemia [8].

For now, the diagnosis of acute appendicitis mainly depends on patient symptoms and serological results, such as white blood cell count (WBC), C-reactive protein (CRP), and neutrophil cells. However, these tests did not present efficient sensitivity and specificity for appendicitis diagnosis [9, 10]. Imaging examinations such as ultrasonography and computed tomography (CT) are also used to assist diagnosis. Ultrasonography has moderate specificity (81%, 95% CI: 78–84%) [11] in detecting acute appendicitis, limiting its diagnostic ability. Though CT examination is more accurate [11], the strong radiation risk and high cost make it unsuitable for wide application. Additionally, several risk scores by combing clinical signs have been developed to improve the predictive ability for appendicitis. The Alvarado score is one of the most well-known scoring systems; it has good sensitivity (99%, 95% CI: 97–99%) but low specificity (43%, 95% CI: 36-51%) [12]. Thus, efficient diagnostic methods for discrimination of appendicitis from other confused acute abdomens are in urgent need.

Urine is a kind of noninvasive, easily attainable clinical sample. With less complexity than serum, urinary proteomics has become an attractive field in biomarker discovery. Current urinary proteomics is mainly applied in diagnostic or prognostic marker discovery for urogenital diseases [13–15] and nonurogenital diseases, such as cholangiocarcinoma [16], coronary artery disease (CAD) [17], heart failure [18], and stroke [19]. Also, for acute appendicitis, candidate urinary biomarkers have been reported, including 5-hydroxyindoleacetic acid (5-HIAA) [20, 21] and leucine-rich *α*-2-glycoprotein (LRG) [22, 23]. However, 5-HIAA was proved to be nonreliable for determining acute appendicitis in afterward studies [24, 25]. LRG had better performance in diagnosing pediatric appendicitis with nonappendicitis, but the diagnostic effects ranged largely in different studies (area under the curve (AUC): 0.63-0.98) [22, 23]. So, the improvement of a urinary proteomic system in acute appendicitis diagnosis is still in great demand.

In this study, a high throughput urinary proteome analysis platform was employed to get deep profiling of urine samples from patients with acute abdomens. The urinary proteomic data were analyzed using several classification strategies for diagnosis of acute appendicitis with other mimic acute abdomen samples. Among these models, a random forest diagnosis model with a panel of 10 urine proteins achieved the best diagnostic result. Without using clinical signs and imaging examinations, the random forest model got an accuracy of 83.6% in the validation dataset, and the sensitivity and specificity were 81.2% and 84.4%, respectively. Hence, our study provides potential urinary markers and efficient model for acute appendicitis diagnosis.

## 2. Materials and Methods

### 2.1. Patient Samples

The study was approved by the Tianjin Baodi Hospital Ethical Review Committee. Urine samples from 134 patients with acute abdomens were collected between 2013 and 2016 from Tianjin Baodi Hospital, including 48 cases with acute appendicitis (AA) and 86 cases with control diseases (cholecystitis and cholelithiasis (CHO), pancreatitis (PAN), gastrointestinal perforation (GP), intestinal obstruction (IO), and other acute abdomens (OTH)). Patients were diagnosed according to blood tests, radiological studies (CT and/or ultrasound), or pathological results after surgery. Patient information was shown in Table 1, and the sample metadata were summarized in [Supplementary-material supplementary-material-1].

### 2.2. Urine Sample Preparation

Midstream urine was obtained from all donors before treatment and stored at -80°C. Urine samples were prepared according to a previously described method ([Supplementary-material supplementary-material-1]) [26]. Briefly, 8.9 ml of urine was centrifuged at 200,000g for 70 min. The pellet was resuspended and heated at 56°C for 30 min to remove uromodulin. After heating, the solution volume was adjusted to 8.9 ml and centrifuged at 200,000g for 70 min to get the final pellet. Then urea buffer (8 M urea, 50 mM Tris, 75 mM NaCl, pH 8.2) was added to resolve the pellet. About 10 *μ*g of protein from each sample was separated by SDS-PAGE in 10% gel. Each gel lane was cut into 3 bands. Each band was cut into small gel pieces, distained with distaining buffer (50% acetonitrile, 50% 50 mM NH_4_HCO_3_), and then washed with 75% acetonitrile and pure water. Gel pieces were digested with trypsin at 37°C overnight.

### 2.3. LC-MS/MS Analysis

The digested peptides were extracted and loaded onto a homemade trap column and an analytical column that both packed with C18 (Dr. Maisch GmbH, Germany). A 75 min gradient was used for online HPLC-MS. Thermo Fisher Orbitrap mass spectrometers were used for measuring all urine samples. LC-MS/MS data were processed using Proteome Discoverer (V1.4, Thermo Fisher) with Mascot algorithm (Mascot V2.3, Matrix Science) against human RefSeq database (2013.07.04). Tryptic peptides of 293T cell lysates were used as quality control samples for evaluation of the instrument reproducibility.

All assigned peptides were filtered with 1% false discovery rate (FDR). Only proteins with ≥2 strict peptides (1% FDR and ion score > 20) were kept for quantification. Intensity-based absolute quantification (iBAQ) algorithm was used for protein quantification. To normalize the differences in loading amounts among samples, the iBAQ value was converted to iFOT (fraction of total, iBAQ value of each protein divided by the sum of all iBAQ values of all proteins in the sample). For better visualization, iFOTs were multiplied by 10^5^ [27]. To eliminate the difference between different groups, the iFOTs were normalized by quantile algorithm [28].

### 2.4. Feature Selection and Construction of Machine-Learning Models

In the discovery dataset, normalized iFOTs were log2 transformed, and the missing values were imputed with a minimal value. For the selection of feature, firstly, AA and CON outliers were screened with the normal reference intervals (RIs) built with 495 normal urine samples [26]. The criterion of outlier is that the iFOT value of a protein is more than 95% CI of normal RI plus 2 folds of interquartile range (IQR). Outlier proteins in each sample were identified and then were further screened depending on the iFOT cutoff value and frequency in the disease group to build disease-related outlier pool. Outlier pool selection standards: (1) the iFOT of the outlier is more than 75% percentile of all the outlier iFOT cutoff values, and it should be outlier in at least 90% percentile of all the outlier frequencies in the AA group or the CON group; (2) if the protein is not expressed in healthy urinary samples, it should be outlier in at least 95% percentile of all the outlier frequencies in the AA group or the CON group. AA outlier pool and CON outlier pool were established, respectively. Then, the nonparametric Wilcoxon test was performed between proteins in the two outlier pools; proteins with *p* value < 0.05 were applied in feature selection algorithm for further screening. Ten proteins were selected and applied in the construction of classification models with different machine-learning algorithms, including random forest (RF), support vector machine (SVM), and naïve Bayes (NB). Leave-one-out cross validation was used in the evaluation of model robustness. To evaluate the prediction effect, the classification models were verified in the independent validation dataset.

### 2.5. Statistical Analysis

R software (version 3.5.3) and RStudio (version 0.99.489) were used in the construction outlier models and machine-learning models, statistical analysis, receiver operating characteristic (ROC) analysis, and plotting. Gene ontology (GO) analysis was performed with DAVID (https://david.ncifcrf.gov/).

## 3. Results and Discussion

### 3.1. Proteomic Profiling of Acute Appendicitis (AA) and Control Acute Abdomen (CON) Samples

**Figure 1:**
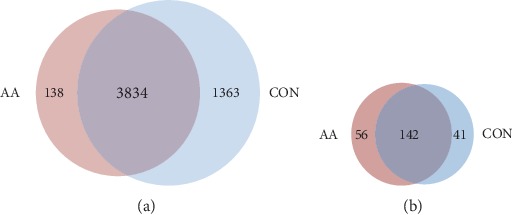
Protein identification in the AA and CON groups. The Venn diagram of the overlaps between (a) total proteins and (b) nonzero proteins identified in the AA and CON groups.

A total of 134 samples passed quality control in protein identification (more than 800 proteins in each sample) were used for data analysis, including 48 AA samples and 86 CON samples (Table 1). High instrument quantification reproducibility was achieved with an average Pearson correlation coefficient of 0.88 between quality control samples. Totally, 5335 proteins were identified and quantified ([Supplementary-material supplementary-material-1]), and an average of 1561 ± 432 proteins was quantified in each sample. Among them, 3834 proteins were identified in both groups. There were 138 unique proteins in the AA group and 1363 unique proteins in the CON group (Figure 1(a)). There were 198 (5.0%) proteins identified in all AA samples, 183 (3.5%) in the CON group, and 142 proteins identified in all the samples (Figure 1(b)). The distribution of proteins showed that the CON group had much more low frequency (<10% frequency) proteins (43.7%) than the AA group (31.4%) ([Supplementary-material supplementary-material-1]). What is more, the highly expressed proteins with more than 90% frequency (AA: 449 proteins; CON: 542 proteins) had a greater proportion than the other groups with more than 20% frequency, suggesting that there were a considerable percentage of “core” urinary proteins widely expressed in different urine samples.

### 3.2. Reliability Demonstration of Profiling Data in Acute Pancreatitis

**Figure 2:**
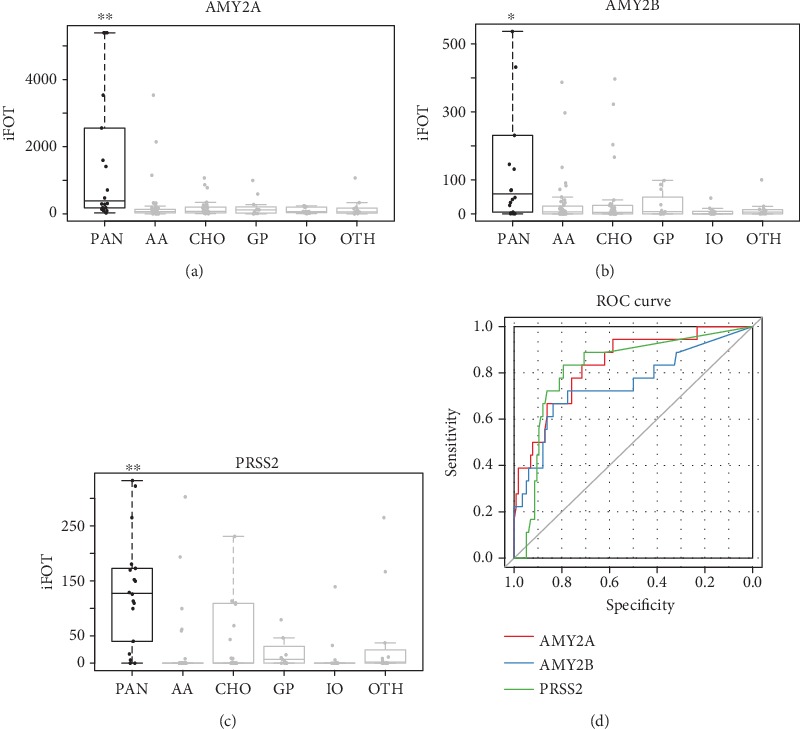
Expression and receiver operating characteristic (ROC) analysis of PAN markers. The abundance of AMY2A (a), AMY2B (b), and PRSS2 (c) in different disease groups (Kruskal-Wallis test, ^∗∗^ means *p* < 0.01, ^∗^ means *p* < 0.05). (d) ROC analysis of AMY2A, AMY2B, and PRSS2, respectively.

To assure whether the urinary proteomic method could be applied in the diagnostic biomarker discovery for nonurogenital disease, we checked the classification ability of our data for acute pancreatitis (PAN) from other acute abdomens because there were commonly accepted reliable urinary markers for pancreatitis. The diagnostic capabilities of the urinary level of amylase (the expression products of AMY2A and AMY2B) and trypsinogen-2 (the expression product of PRSS2) had been proved in many researches [29, 30]. We found that AMY2A, AMY2B, and PRSS2 were significantly highly expressed in urine samples from pancreatitis patients compared with those from other acute abdomen patients (Figures 2(a)–2(c), Kruskal-Wallis test, *p* < 0.05). The AUCs of the three proteins between pancreatitis and the rest of the acute abdomen samples were all above 0.75 (Figure 2(d), Table 2). The AUC of AMY2A was 0.83, which was the best one among these three proteins, showing its outstanding ability in diagnosing pancreatitis. This result demonstrated that the urinary proteome system could produce reliable datasets, and proteins derived from nonurogenital tissues could be detected and beneficial in disease diagnosis.

### 3.3. Feature Selection for Classification of AA and CON

Based on the type of mass spectrometers (MS), the dataset was divided into discovery dataset and independent validation dataset ([Supplementary-material supplementary-material-1]). In the discovery dataset, a total of 73 urine samples including acute appendicitis (AA, *n* = 32) and control acute abdomen (CON, *n* = 41) specimens that processed with multiple MS types were used for feature selection and classification model construction. The independent validation dataset consisted of 16 AA and 45 CON specimens, which were all produced by the same MS type (Orbitrap-QE-Plus).

**Figure 3:**
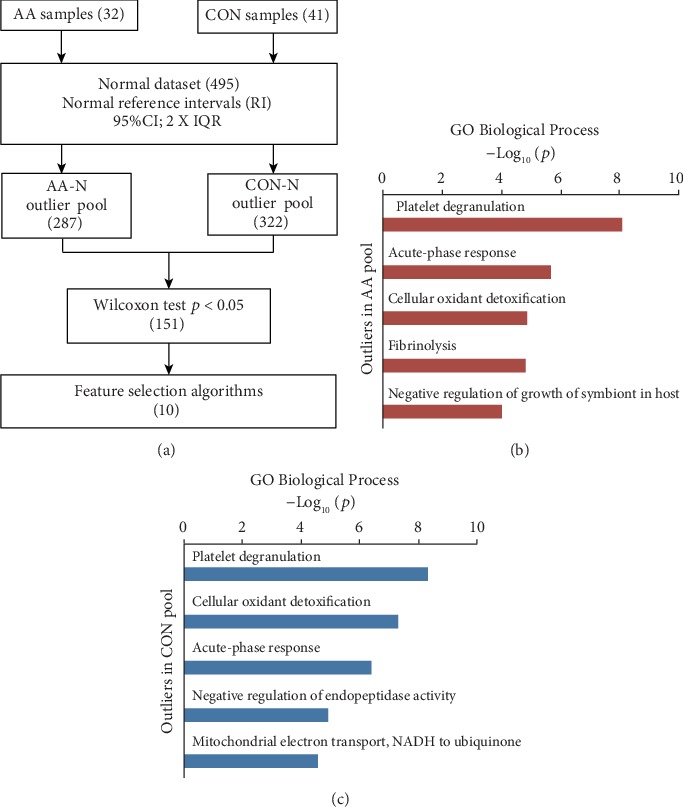
Feature selection workflow (a) and GO Biological Process analysis of outlier proteins in AA (b) and CON (c) outlier pools.

In order to select the features for classification (Figure 3(a)), AA outliers and CON outliers were identified, respectively, in the discovery dataset against a normal urine database including 495 samples. Outliers were proteins with expression that is higher than 95% confidence interval (CI) of reference interval (RI) plus 2 folds of interquartile range (IQR) of RI. The frequently detected outliers could be considered as markers to reveal changes under pathological conditions. With further screening by frequency (shown in Methods), 287 proteins were selected as AA outliers, and 322 were CON outliers ([Supplementary-material supplementary-material-1]).

For the above two outlier pools, Gene ontology (GO) analysis was conducted (Figures 3(b), 3(c), and [Supplementary-material supplementary-material-1]). Among the top 5 processes, three items including platelet degranulation, acute-phase response, and cellular oxidant detoxification were enriched both in AA outliers and CON outliers (Figures 3(b) and 3(c)). For proteins enriched in acute-phase response (ranked second and third in AA outliers and CON outliers, respectively), eight proteins were shared by AA and CON; one protein (ITIH4, inter-alpha-trypsin inhibitor heavy chain family member 4) was CON-specific. Hence, in order to distinguish different diseases, specific proteins in outliers should be further filtered as classification features. The 151 significantly differential proteins between the two groups (nonparametric Wilcoxon test, *p* value < 0.05, [Supplementary-material supplementary-material-1]) were used for feature selection algorithm. Ten proteins (LYVE1: lymphatic vessel endothelial hyaluronan receptor 1; AHCYL1: adenosylhomocysteinase-like 1; APOC1: apolipoprotein C1; SECTM1: secreted and transmembrane 1; SLC31A1: solute carrier family 31 member 1; ITGA6: integrin subunit alpha 6; SLC35F2: solute carrier family 35 member F2; GPX3: glutathione peroxidase 3; TMEM14C: transmembrane protein 14C; SLC47A2: solute carrier family 47 member 2) were further selected for the establishment of classification models, and their expressions were shown in [Supplementary-material supplementary-material-1].

### 3.4. Construction and Evaluation of Classification Models

**Figure 4:**
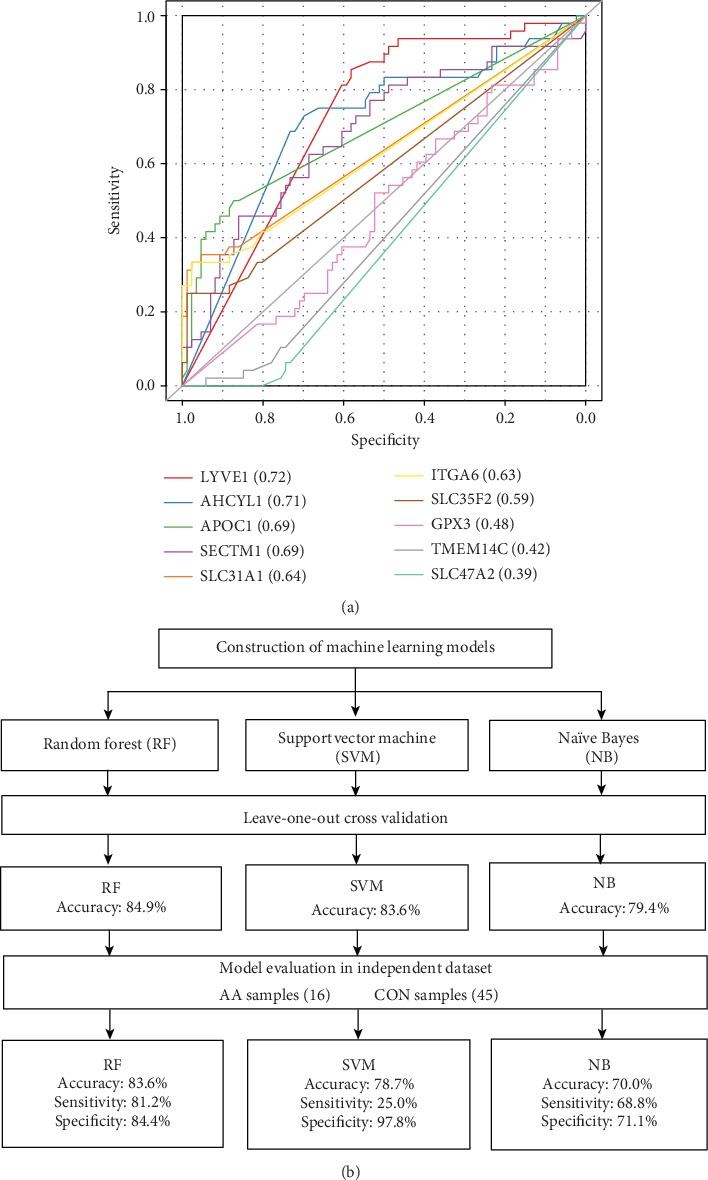
Construction and evaluation of diagnosis models for AA and CON. (a) ROC analysis of one-feature-based models; (b) classification model construction by 3 machine-learning algorithms: random forest (RF), support vector machine (SVM), and Naïve Bayes (NB) in the discovery dataset, and their performance evaluation by leave-one-out cross validation and the independent test.

However, AUCs of the ten single-feature-based classification models were all less than 0.75 (Figure 4(a)), indicating the limitation of single feature in classification. Aiming at model optimization, machine-learning algorithms were further adopted to integrate multiple features for model construction, including random forest (RF), support vector machine (SVM), and naïve Bayes (NB) (Figure 4(b)). The robustness of these 3 models was evaluated by leave-one-out cross validation, and the accuracies of the 3 models were 84.9%, 83.6%, and 79.4%, respectively. In the independent validation dataset, 16 AA and 45 CON specimens were included. The RF model could correctly distinguish 83.6% of samples (sensitivity: 81.2%, specificity: 84.4%), SVM model had an accuracy of 78.7% (sensitivity: 25.0%, specificity: 97.8%), and NB had 70.5% (sensitivity: 68.8%, specificity: 71.1%), respectively. The RF model achieved the best performance among the 3 models and showed advantage in differential classification.

## 4. Discussion

Acute appendicitis is one of the most significant acute abdomen diseases in clinic, but the diagnosis of acute appendicitis is still difficult. In our study, a urinary proteomic system was applied to discover urinary markers for differential diagnosis of AA with other acute abdomens. A panel of 10 proteins integrated by the RF model achieves 84.9% of accuracy in leave-one-out cross validation and 83.6% in the independent validation dataset, which was better than the reported results of WBC (sensitivity 62%, specificity 75%) and CRP (sensitivity 57%, specificity 87%) [10]. Notably, without relying on any clinical sign, this RF model using urine markers showed high specificity in AA diagnosis, which was helpful in reducing negative appendectomy.

Among the 10 features in this diagnostic model, two features had AUCs higher than 0.7, including LYVE1 (lymphatic vessel endothelial hyaluronan receptor 1) and AHCYL1 (adenosylhomocysteinase-like 1). LYVE1 is an immunity-related factor that may play a role in the migration of immune cells, and the loss of LYVE1 is a sign of activation of inflammation [31, 32]. The expression of LYVE1 is lower in the AA group than in the CON group. Since the appendix is also an immune organ, this may indicate that inflammation is more easily activated in AA. AHCYL1 is upregulated in the CON group, maybe because it acts as a regulator of fluid secretion [33], which is important for the secretary organs in the CON group. APOC1 (apolipoprotein C1) is a feature that upregulated in the AA group. APOC1 can bind to lipopolysaccharide (LPS) on the outer-membrane of gram-negative bacteria to present LPS to macrophages, thereby stimulating the inflammatory response [34]. The upregulation of APOC1 might reflect the existence of bacterial infection in AA than the diseases in the CON group, such as cholecystitis and pancreatitis.

In this study, we found that the urinary protein panel and the random forest model were powerful in the diagnosis of acute appendicitis with the other acute abdomens. The advantages of random forest algorithm, such as nonoverfitting, robustness to noise, make it a robust tool in classification. Our results also demonstrated the potential of urinary protein in the diagnosis of nonurogenital diseases, as the change of acute pancreatitis biomarkers could be well detected in different disease groups. With the relatively high specificity, our result might provide beneficial supplement to the clinical auxiliary diagnosis of acute appendicitis. However, there are still areas that need further improvement. Firstly, this urinary diagnostic model should be further validated in a larger number of samples. Secondly, for facilitation in potential clinical application, the feature proteins need to be absolutely quantified. Except for the immunological methods, the MS-based method for quantification is becoming popular, with the increasingly high-throughput detection of target proteins independent on antibodies. Lastly, since urine is rich of small metabolites, the combination of urinary protein makers with metabolites might contribute to improving diagnostic efficiency.

## 5. Conclusions

In summary, a urinary proteomic system was applied to discover urinary markers for differential diagnosis of AA with other acute abdomens. A panel of 10 proteins integrated by the RF model achieves 84.9% of accuracy in leave-one-out cross validation and 83.6% in the independent validation dataset. Without relying on any clinical sign, this RF model using urine markers showed high specificity in AA diagnosis, which was helpful in reducing negative appendectomy, indicating the potential of noninvasive urinary markers in clinical application.

## Figures and Tables

**Table 1:** Information of patients with acute appendicitis and other control diseases.

Diseases	Discovery set (*n* = 73)			Validation set (*n* = 61)		
	Cases	Gender (M/F)	Age (year)	Cases	Gender (M/F)	Age (year)
Acute appendicitis (AA, *n* = 48)	32	14/18	38.5 ± 17.9	16	9/7	34.2 ± 15.2
Control acute abdomens (CON, *n* = 86)						
Cholecystitis and gallstones (CHO)	17	5/12	56.9 ± 17.0	15	9/6	62.7 ± 16.3
Pancreatitis (PAN)	5	0/5	42.0 ± 13.9	13	10/3	48.0 ± 13.9
Gastrointestinal perforation (GP)	6	4/2	67.2 ± 17.6	5	5/0	66.8 ± 14.1
Intestinal obstruction (IO)	9	4/5	60.3 ± 15.9	4	1/3	58.0 ± 13.0
Other abdomens (OTH)	4	0/4	34.2 ± 12.3	8	2/6	47.2 ± 25.5
Total	41	13/28	55.1 ± 18.2	45	14/17	56.6 ± 17.2

**Table 2:** AUC, sensitivity, and specificity of PAN marker proteins in diagnosing PAN with other acute abdomens.

Proteins	AUC	Sensitivity	Specificity
AMY2A	0.83	83.3%	71.5%
AMY2B	0.75	66.7%	83.6%
PRSS2	0.82	83.3%	79.3%

## Data Availability

The data used to support the findings of this study are available from the corresponding authors upon request.

## Supplementary Materials

Supplementary 1 Figure S1: the overview of experimental workflow for the urinary proteomic method.

Supplementary 2 Figure S2: the distribution of proteins with different frequencies in the AA and CON groups.

Supplementary 3 Figure S3: GO enrichment analysis of outliers in the AA and CON pools, respectively. (A) Cellular component analysis of the AA outlier pool. (B) Cellular component analysis of the CON outlier pool. (C) Molecular function analysis of the AA outlier pool. (D) Molecular function analysis of the CON outlier pool.

Supplementary 4 Figure S4: relative abundance of 10 feature proteins in the AA and CON groups. The Wilcoxon test was performed between the AA and CON groups, ^∗∗^ means *p* < 0.01, ^∗^ means *p* < 0.05.

Supplementary 5 Table S1: metadata of urine samples that analyzed in this study, including the sample ID, group label, dataset type, gender age, disease status, sampled date, sampled hospital, and MS instrument.

Supplementary 6 Table S2: sheet 1: relative abundances (iFOT) of discovery dataset; sheet 2: relative abundances (iFOT) of validation dataset; sheet 3: list of 287 outlier proteins in the AA group; sheet 4: list of 322 outlier proteins in the CON groups; sheet 5: list of 151 outlier proteins that are significantly different between AA and CON groups.
