# Supplementary material for: Discovery of Urinary Proteomic Signature for Differential Diagnosis of Acute Appendicitis
Source: Biomed Res Int. 2020 Apr 4;2020:3896263. doi: 10.1155/2020/3896263 (PMC7165319; doi:10.1155/2020/3896263)
Supplement: Supplementary 1 — Figure S1: the overview of experimental workflow for the urinary proteomic method. [file 3896263.f1.pdf]

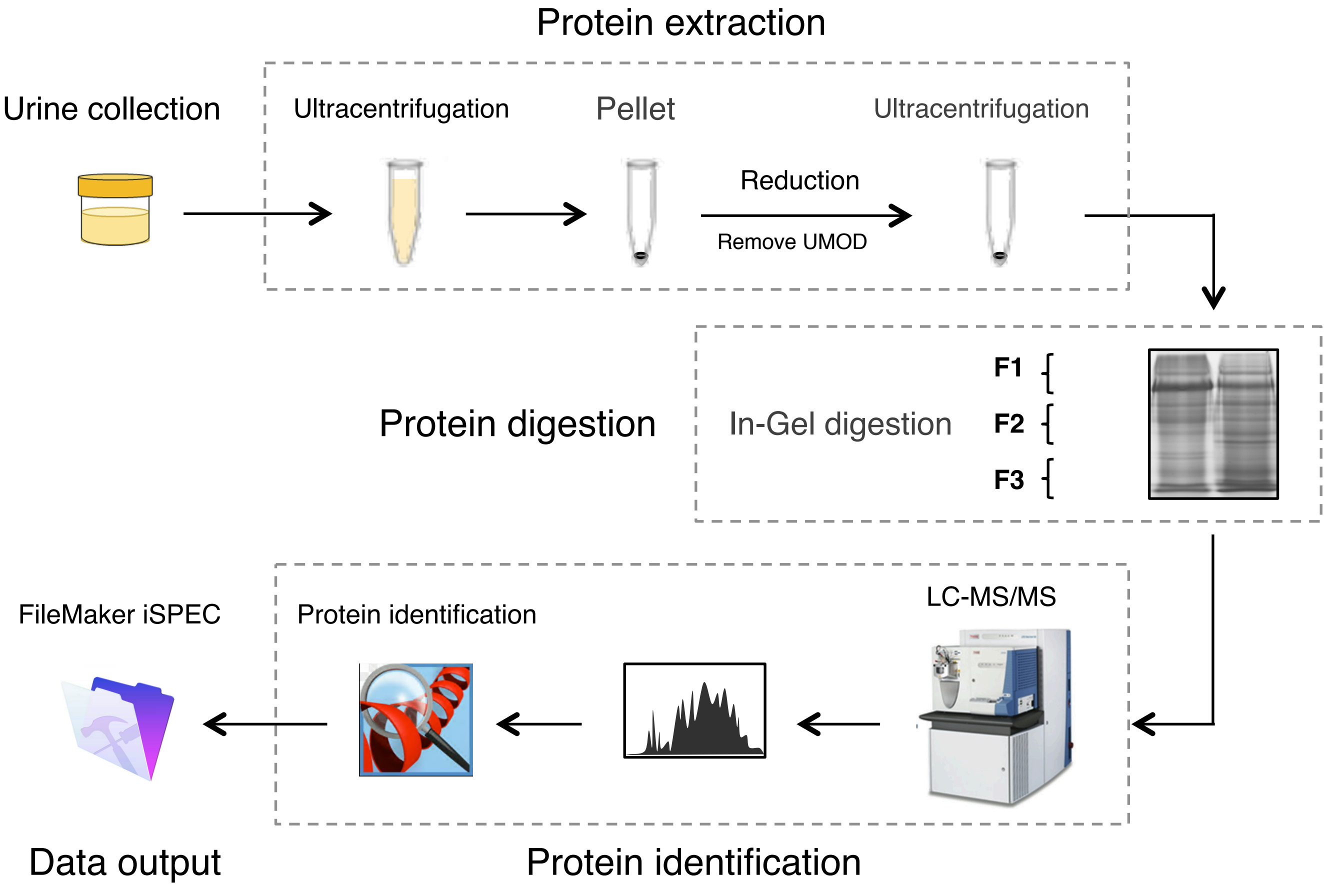
Protein extraction
Urine collection
Ultracentrifugation
Pellet
Ultracentrifugation
Reduction
Remove UMOD
Protein digestion
In-Gel digestion
F1
F2
F3
FileMaker iSPEC
Protein identification
LC-MS/MS
Data output
Protein identification
