# Supplementary figures and images for: Discovery of Urinary Proteomic Signature for Differential Diagnosis of Acute Appendicitis
Source: Biomed Res Int. 2020 Apr 4;2020:3896263. doi: 10.1155/2020/3896263 (PMC7165319; doi:10.1155/2020/3896263)

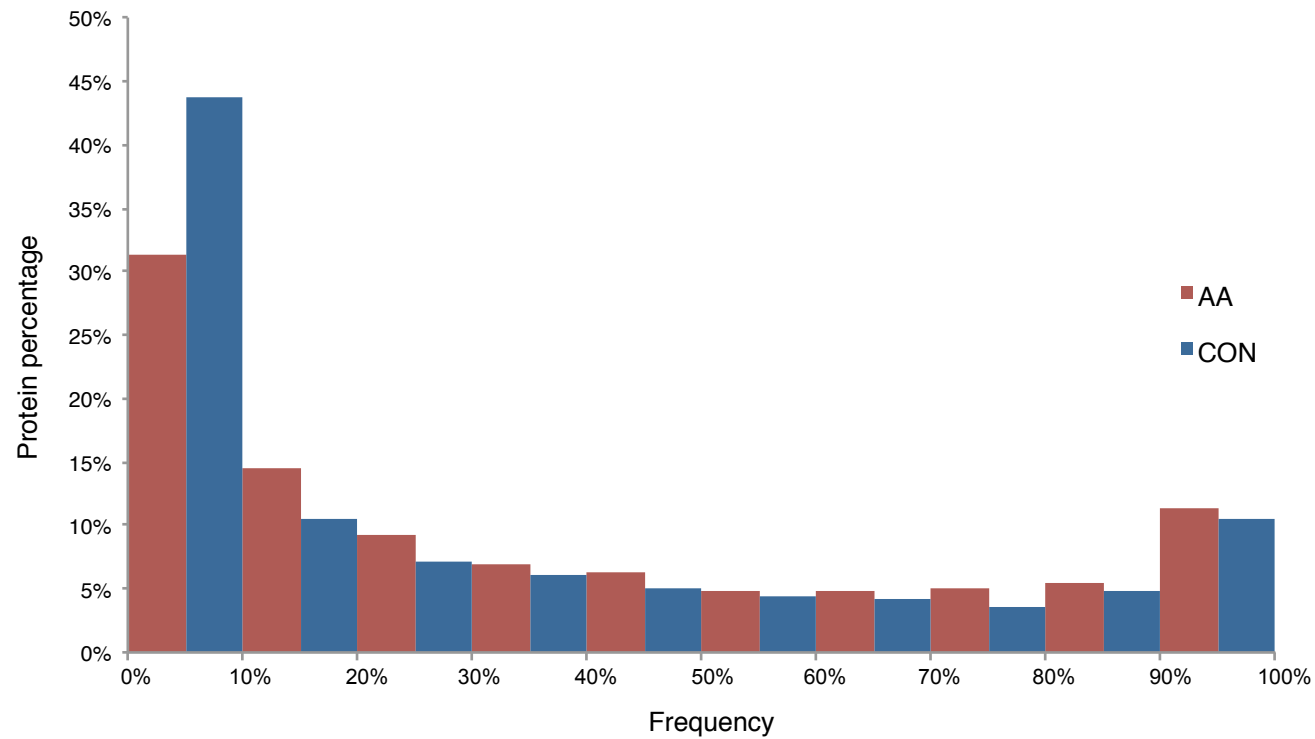

Supplement: Supplementary 2 — Figure S2: the distribution of proteins with different frequencies in the AA and CON groups. [file 3896263.f2.pdf]

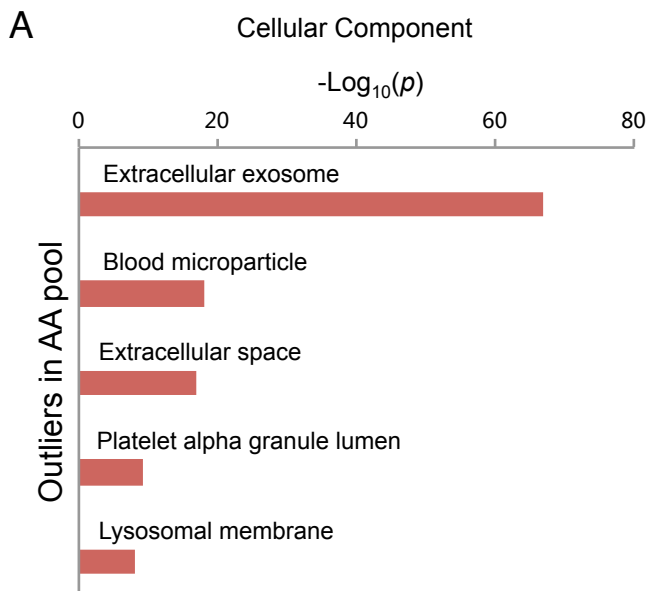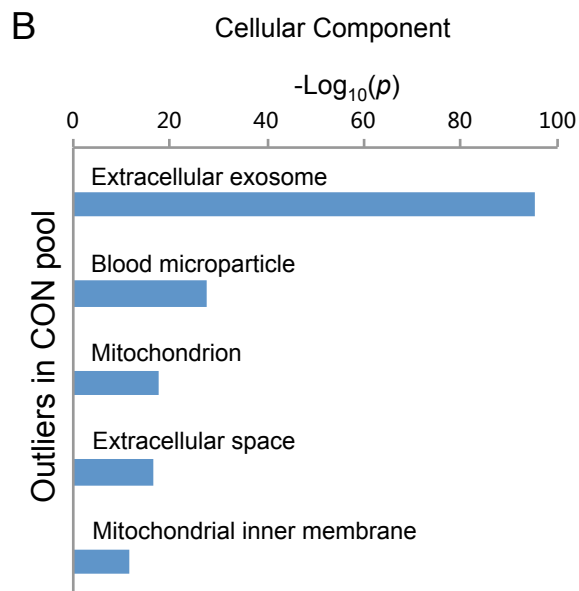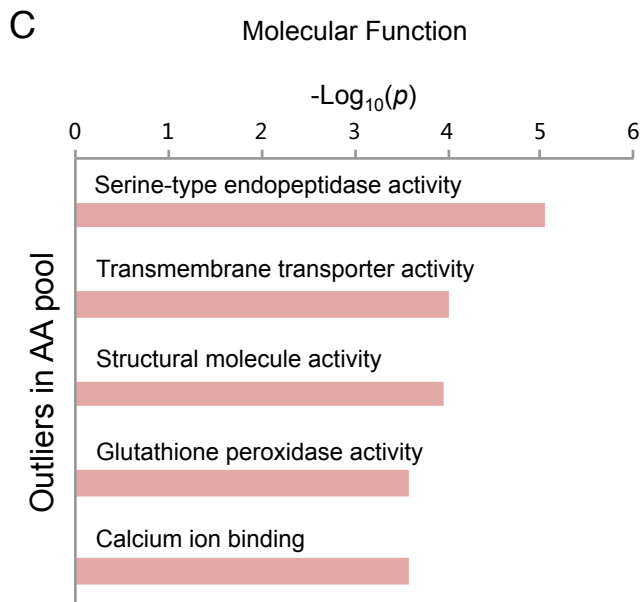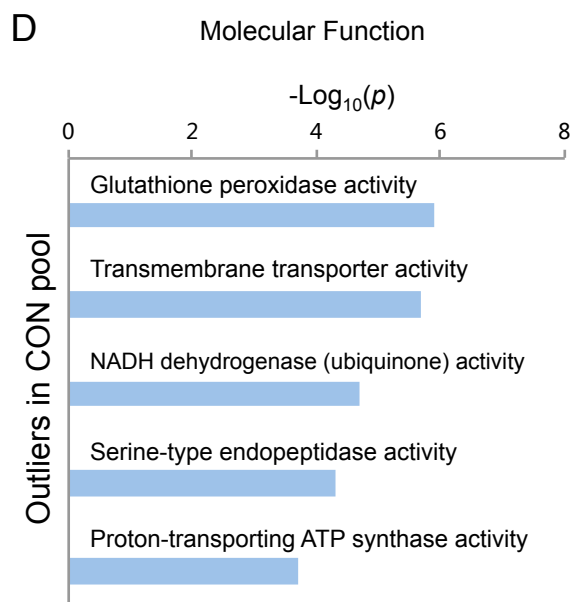

Supplement: Supplementary 3 — Figure S3: GO enrichment analysis of outliers in the AA and CON pools, respectively. (A) Cellular component analysis of the AA outlier pool. (B) Cellular component analysis of the CON outlier pool. (C) Molecular function analysis of the AA outlier pool. (D) Molecular function analysis of the CON outlier pool. [file 3896263.f3.pdf]

IYVE1

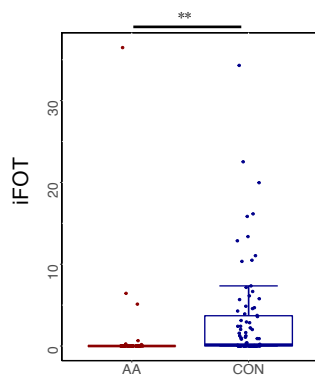

AHCYL1

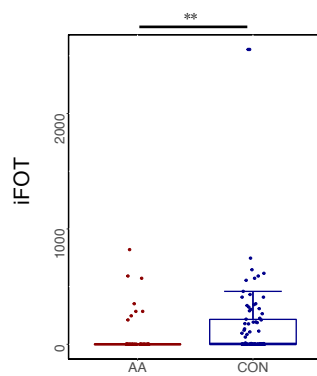

APOC1

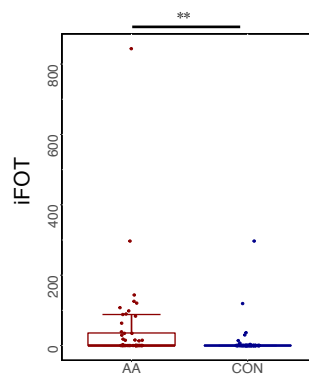

SECTM1

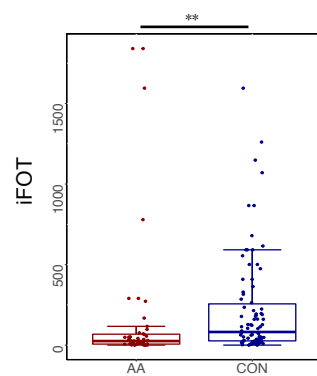

SLC31A1

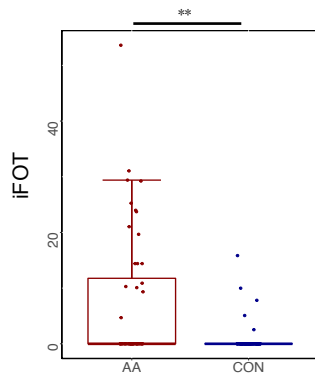

ITGA6

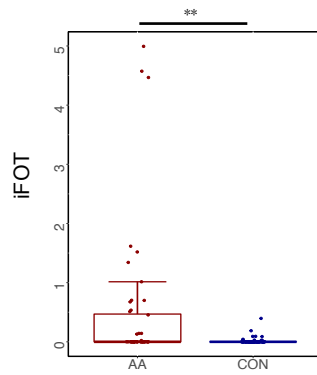

SLC35F2

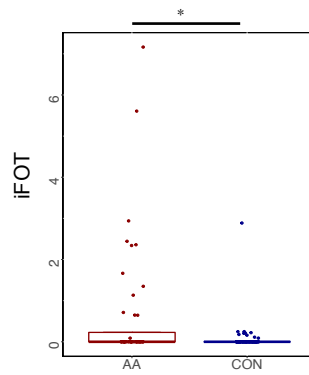

GPX3

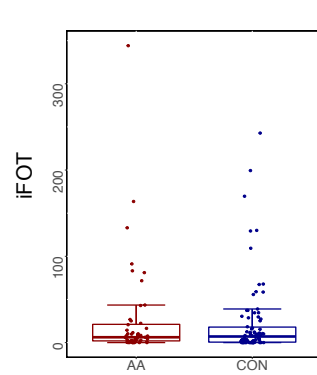

TMEM14C

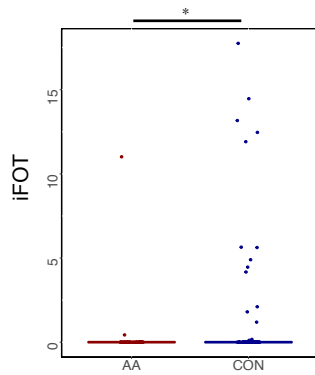

SLC47A2

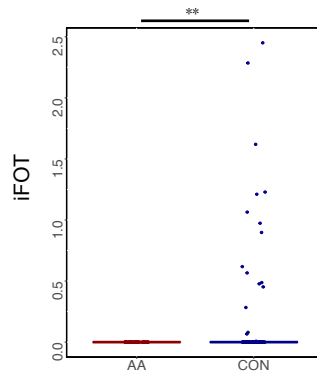

Supplement: Supplementary 4 — Figure S4: relative abundance of 10 feature proteins in the AA and CON groups. The Wilcoxon test was performed between the AA and CON groups, ∗∗ means p < 0.01, ∗ means p < 0.05. [file 3896263.f4.pdf]
